# Erratum

**DOI:** 10.1016/j.cjcpc.2023.01.002

**Published:** 2023-01-25

The publisher regrets that Editor disclaimer statements were not included in the published version of the following articles that appeared in previous issues of *CJC Pediatric and Congenital Heart Disease*.

The appropriate disclaimer statements are included below.

1. “Association of Acute Anti-inflammatory Treatment With Medium-term Outcomes for Coronary Artery Aneurysms in Kawasaki Disease” [CJC Pediatric and Congenital Heart Disease, Volume 1, Issue 4, 2022, Pages 174-183] https://doi.org/10.1016/j.cjcpc.2022.05.007

Editorial Disclaimer:

Given his role as Editor in Chief, Kevin Harris, had no involvement in the peer review of this article and has no access to information regarding its peer review. Full responsibility for the editorial process for this article was delegated to Stanley Nattel.

Given their role as Associate Editors, Frederic Dallaire, Andrew Mackie, and Brian McCrindle had no involvement in the peer review of this article and have no access to information regarding its peer review.

2. “Socioeconomic Status and Kawasaki Disease Outcomes in a Single-Payer Health Care System” [CJC Pediatric and Congenital Heart Disease, Volume 1, Issue 6, 2022, Pages 248-252] https://doi.org/10.1016/j.cjcpc.2022.10.007

Editorial Disclaimer: Given his role as Associate Editor, Brian McCrindle had no involvement in the peer review of this article and has no access to information regarding its peer review.

3. “Reduced Physical Activity During COVID-19 in Children With Congenital Heart Disease: A Longitudinal Analysis” [CJC Pediatric and Congenital Heart Disease, Volume 1, Issue 5, 2022, Pages 219-225] https://doi.org/10.1016/j.cjcpc.2022.05.006

Editorial Disclaimer: Given his role as Editor in Chief, Kevin Harris, had no involvement in the peer review of this article and has no access to information regarding its peer review. Full responsibility for the editorial process for this article was delegated to Stanley Nattel.

4. “A Survey of Immunization Practices in Patients With Congenital Heart Disease” [CJC Pediatric and Congenital Heart Disease, Volume 1, Issue 2, 2022, Pages 74-79, ISSN 2772-8129] https://doi.org/10.1016/j.cjcpc.2021.12.003

Editorial Disclaimer: Given his role as Editor in Chief, Kevin Harris, had no involvement in the peer review of this article and has no access to information regarding its peer review. Full responsibility for the editorial process for this article was delegated to Michelle Graham.

5. “Physical Activity in Paediatric Long QT Syndrome Patients” [CJC Pediatric and Congenital Heart Disease, Volume 1, Issue 2, 2022, Pages 80-85] https://doi.org/10.1016/j.cjcpc.2021.12.001

Editorial Disclaimer: Given his role as Editor in Chief, Kevin Harris, had no involvement in the peer review of this article and has no access to information regarding its peer review. Full responsibility for the editorial process for this article was delegated to Michelle Graham.

